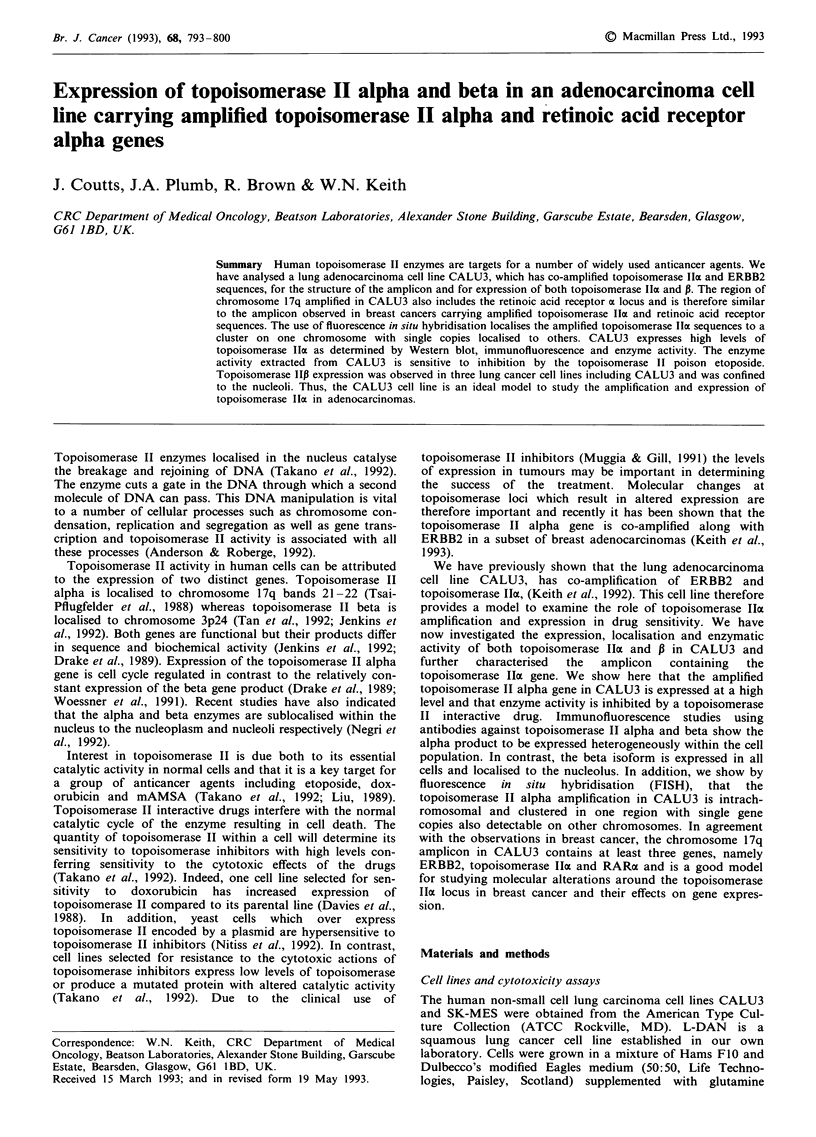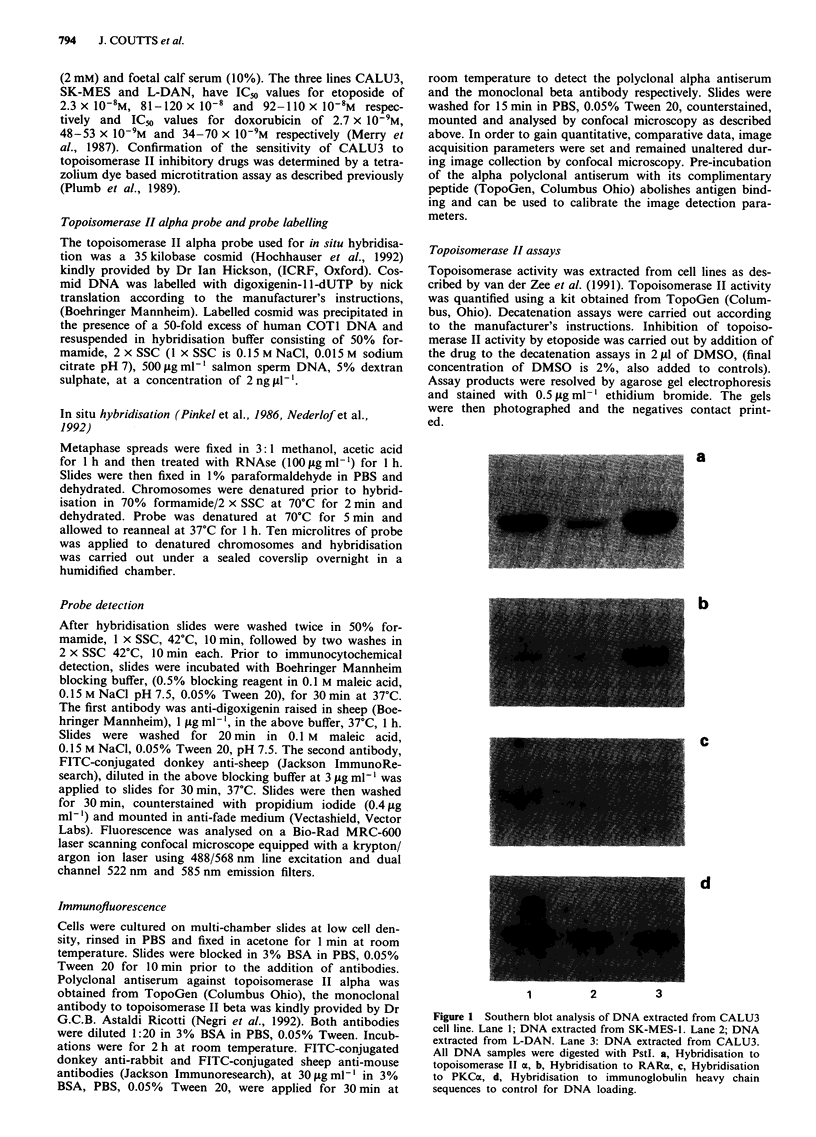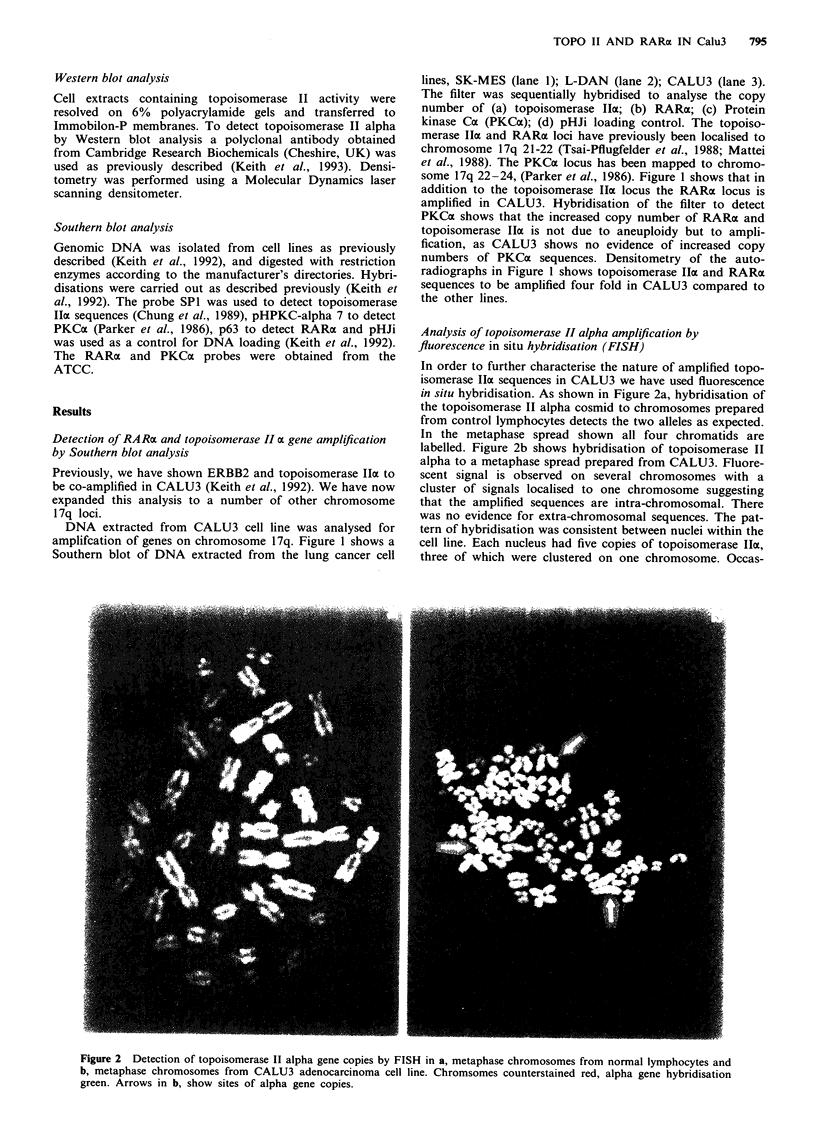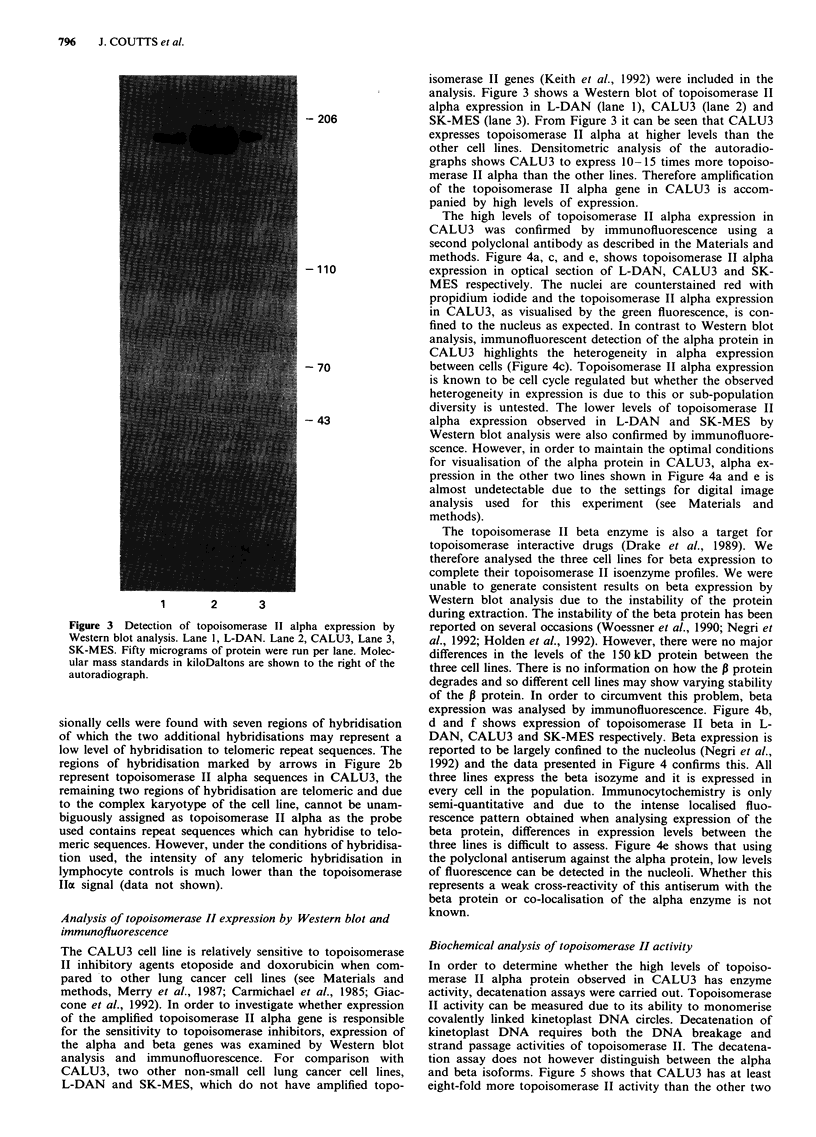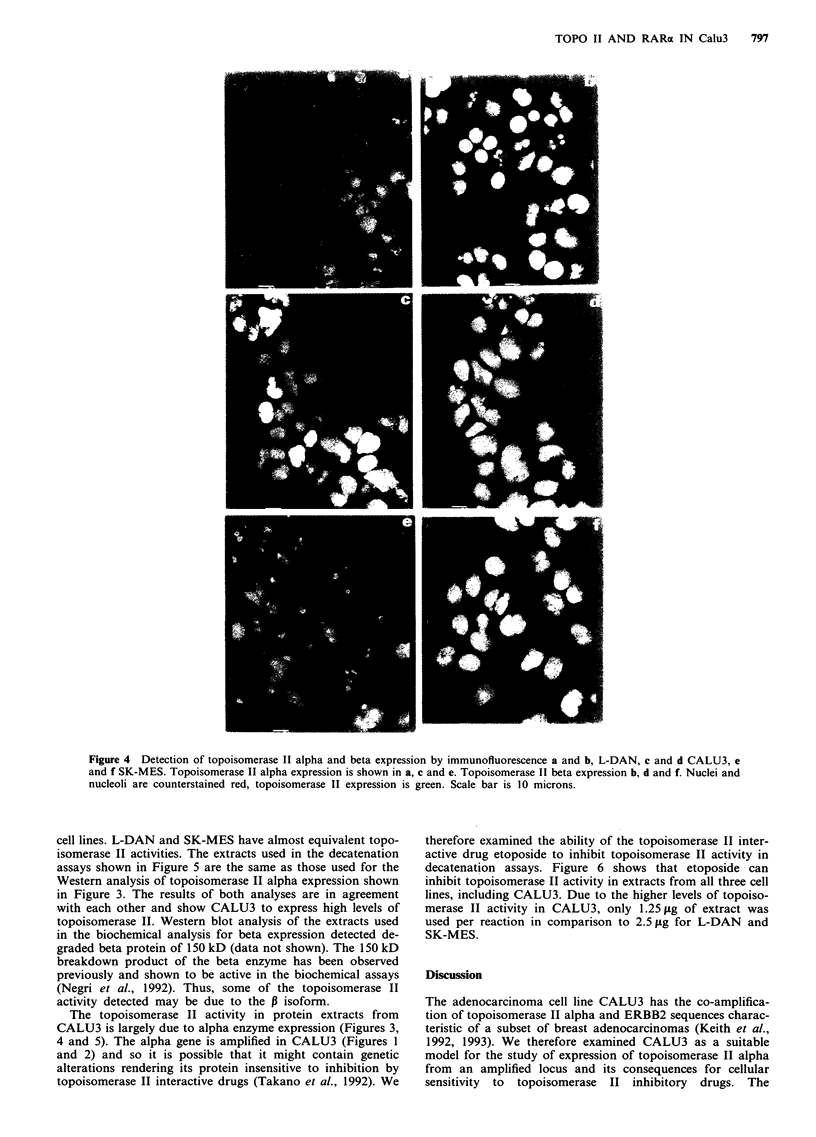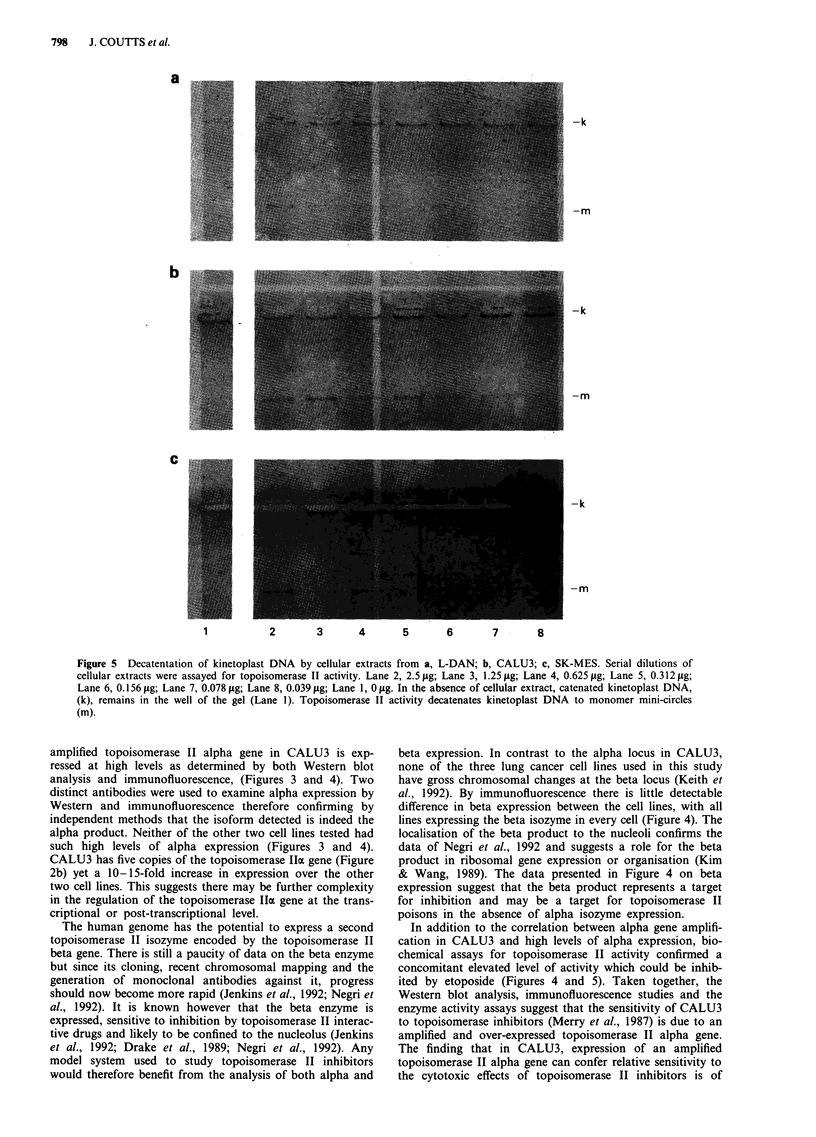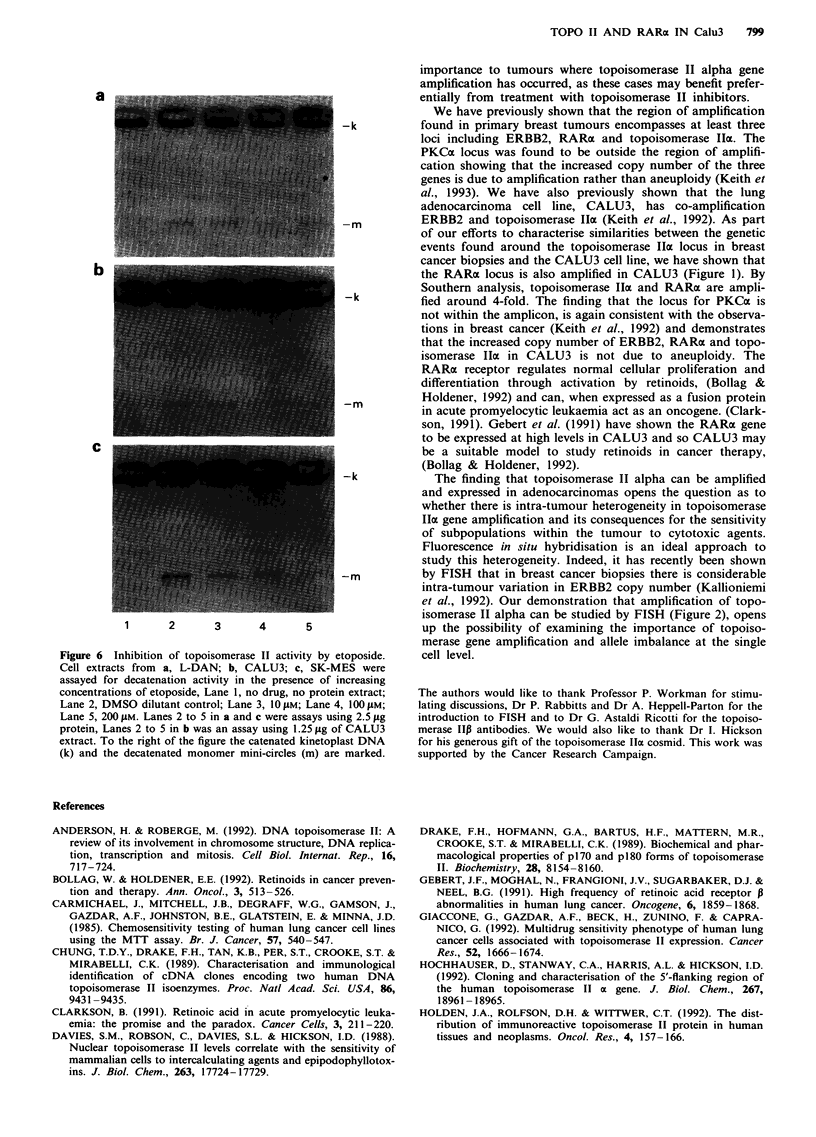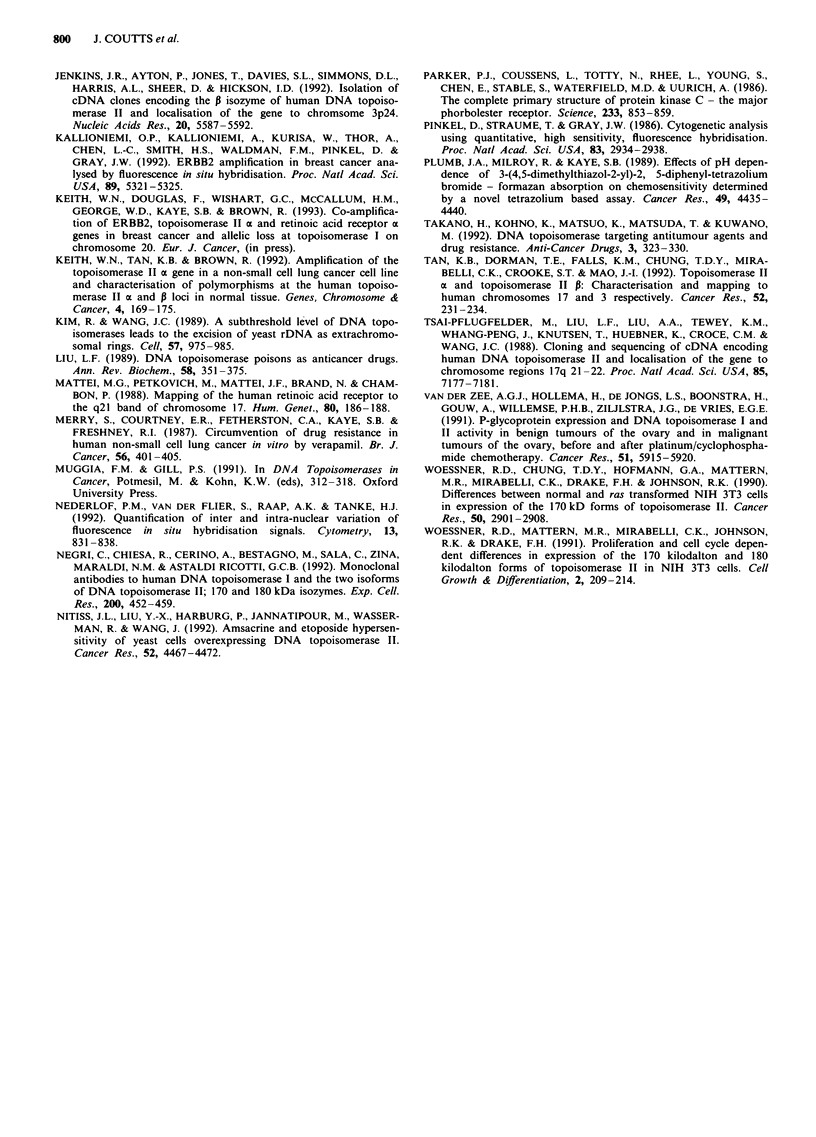# Expression of topoisomerase II alpha and beta in an adenocarcinoma cell line carrying amplified topoisomerase II alpha and retinoic acid receptor alpha genes.

**DOI:** 10.1038/bjc.1993.430

**Published:** 1993-10

**Authors:** J. Coutts, J. A. Plumb, R. Brown, W. N. Keith

**Affiliations:** CRC Department of Medical Oncology, Beatson Laboratories, Bearsden, Glasgow, UK.

## Abstract

**Images:**


Br. J. Cancer (1993), 68, 793–800 © Macmillan Press Ltd., 1993

### Expression of topoisomerase II alpha and beta in an adenocarcinoma cell line carrying amplified topoisomerase II alpha and retinoic acid receptor alpha genes

J. Coutts, J.A. Plumb, R. Brown & W.N. Keith

*CRC Department of Medical Oncology, Beatson Laboratories, Alexander Stone Building, Garscube Estate, Bearsden, Glasgow, G61 1BD, UK.*

**Summary** Human topoisomerase II enzymes are targets for a number of widely used anticancer agents. We have analysed a lung adenocarcinoma cell line CALU3, which has co-amplified topoisomerase IIα and ERBB2 sequences, for the structure of the amplicon and for expression of both topoisomerase IIα and β. The region of chromosome 17q amplified in CALU3 also includes the retinoic acid receptor α locus and is therefore similar to the amplicon observed in breast cancers carrying amplified topoisomerase IIα and retinoic acid receptor sequences. The use of fluorescence *in situ* hybridisation localises the amplified topoisomerase IIα sequences to a cluster on one chromosome with single copies localised to others. CALU3 expresses high levels of topoisomerase IIα as determined by Western blot, immunofluorescence and enzyme activity. The enzyme activity extracted from CALU3 is sensitive to inhibition by the topoisomerase II poison etoposide. Topoisomerase IIβ expression was observed in three lung cancer cell lines including CALU3 and was confined to the nucleoli. Thus, the CALU3 cell line is an ideal model to study the amplification and expression of topoisomerase IIα in adenocarcinomas.

Topoisomerase II enzymes localised in the nucleus catalyse the breakage and rejoining of DNA (Takano *et al.*, 1992). The enzyme cuts a gate in the DNA through which a second molecule of DNA can pass. This DNA manipulation is vital to a number of cellular processes such as chromosome condensation, replication and segregation as well as gene transcription and topoisomerase II activity is associated with all these processes (Anderson & Roberge, 1992).

Topoisomerase II activity in human cells can be attributed to the expression of two distinct genes. Topoisomerase II alpha is localised to chromosome 17q bands 21–22 (Tsai-Pflugfelder *et al.*, 1988) whereas topoisomerase II beta is localised to chromosome 3p24 (Tan *et al.*, 1992; Jenkins *et al.*, 1992). Both genes are functional but their products differ in sequence and biochemical activity (Jenkins *et al.*, 1992; Drake *et al.*, 1989). Expression of the topoisomerase II alpha gene is cell cycle regulated in contrast to the relatively constant expression of the beta gene product (Drake *et al.*, 1989; Woessner *et al.*, 1991). Recent studies have also indicated that the alpha and beta enzymes are sublocalised within the nucleus to the nucleoplasm and nucleoli respectively (Negri *et al.*, 1992).

Interest in topoisomerase II is due both to its essential catalytic activity in normal cells and that it is a key target for a group of anticancer agents including etoposide, doxorubicin and mAMSA (Takano *et al.*, 1992; Liu, 1989). Topoisomerase II interactive drugs interfere with the normal catalytic cycle of the enzyme resulting in cell death. The quantity of topoisomerase II within a cell will determine its sensitivity to topoisomerase inhibitors with high levels conferring sensitivity to the cytotoxic effects of the drugs (Takano *et al.*, 1992). Indeed, one cell line selected for sensitivity to doxorubicin has increased expression of topoisomerase II compared to its parental line (Davies *et al.*, 1988). In addition, yeast cells which over express topoisomerase II encoded by a plasmid are hypersensitive to topoisomerase II inhibitors (Nitiss *et al.*, 1992). In contrast, cell lines selected for resistance to the cytotoxic actions of topoisomerase inhibitors express low levels of topoisomerase or produce a mutated protein with altered catalytic activity (Takano *et al.*, 1992). Due to the clinical use of topoisomerase II inhibitors (Muggia & Gill, 1991) the levels of expression in tumours may be important in determining the success of the treatment. Molecular changes at topoisomerase loci which result in altered expression are therefore important and recently it has been shown that the topoisomerase II alpha gene is co-amplified along with ERBB2 in a subset of breast adenocarcinomas (Keith *et al.*, 1993).

We have previously shown that the lung adenocarcinoma cell line CALU3, has co-amplification of ERBB2 and topoisomerase IIα, (Keith *et al.*, 1992). This cell line therefore provides a model to examine the role of topoisomerase IIα amplification and expression in drug sensitivity. We have now investigated the expression, localisation and enzymatic activity of both topoisomerase IIα and β in CALU3 and further characterised the amplicon containing the topoisomerase IIα gene. We show here that the amplified topoisomerase II alpha gene in CALU3 is expressed at a high level and that enzyme activity is inhibited by a topoisomerase II interactive drug. Immunofluorescence studies using antibodies against topoisomerase II alpha and beta show the alpha product to be expressed heterogeneously within the cell population. In contrast, the beta isoform is expressed in all cells and localised to the nucleolus. In addition, we show by fluorescence *in situ* hybridisation (FISH), that the topoisomerase II alpha amplification in CALU3 is intrachromosomal and clustered in one region with single gene copies also detectable on other chromosomes. In agreement with the observations in breast cancer, the chromosome 17q amplicon in CALU3 contains at least three genes, namely ERBB2, topoisomerase IIα and RARα and is a good model for studying molecular alterations around the topoisomerase IIα locus in breast cancer and their effects on gene expression.

### Materials and methods

### *Cell lines and cytotoxicity assays*

The human non-small cell lung carcinoma cell lines CALU3 and SK-MES were obtained from the American Type Culture Collection (ATCC Rockville, MD). L-DAN is a squamous lung cancer cell line established in our own laboratory. Cells were grown in a mixture of Hams F10 and Dulbecco's modified Eagles medium (50:50, Life Technologies, Paisley, Scotland) supplemented with glutamine

Correspondence: W.N. Keith, CRC Department of Medical Oncology, Beatson Laboratories, Alexander Stone Building, Garscube Estate, Bearsden, Glasgow, G61 1BD, UK.
Received 15 March 1993; and in revised form 19 May 1993.

(2 mM) and foetal calf serum (10%). The three lines CALU3, SK-MES and L-DAN, have $IC_{50}$ values for etoposide of $2.3 \times 10^{-8}$M, $81-120 \times 10^{-8}$ and $92-110 \times 10^{-8}$M respectively and $IC_{50}$ values for doxorubicin of $2.7 \times 10^{-9}$M, $48-53 \times 10^{-9}$M and $34-70 \times 10^{-9}$M respectively (Merry *et al.*, 1987). Confirmation of the sensitivity of CALU3 to topoisomerase II inhibitory drugs was determined by a tetrazolium dye based microtitration assay as described previously (Plumb *et al.*, 1989).

*Topoisomerase II alpha probe and probe labelling*

The topoisomerase II alpha probe used for *in situ* hybridisation was a 35 kilobase cosmid (Hochhauser *et al.*, 1992) kindly provided by Dr Ian Hickson, (ICRF, Oxford). Cosmid DNA was labelled with digoxigenin-11-dUTP by nick translation according to the manufacturer's instructions, (Boehringer Mannheim). Labelled cosmid was precipitated in the presence of a 50-fold excess of human COT1 DNA and resuspended in hybridisation buffer consisting of 50% formamide, 2 × SSC (1 × SSC is 0.15 M NaCl, 0.015 M sodium citrate pH 7), 500 $\mu$g ml$^{-1}$ salmon sperm DNA, 5% dextran sulphate, at a concentration of 2 ng $\mu$l$^{-1}$.

In situ *hybridisation (Pinkel* et al., *1986, Nederlof* et al., *1992)*

Metaphase spreads were fixed in 3:1 methanol, acetic acid for 1 h and then treated with RNAse (100 $\mu$g ml$^{-1}$) for 1 h. Slides were then fixed in 1% paraformaldehyde in PBS and dehydrated. Chromosomes were denatured prior to hybridisation in 70% formamide/2 × SSC at 70°C for 2 min and dehydrated. Probe was denatured at 70°C for 5 min and allowed to reanneal at 37°C for 1 h. Ten microlitres of probe was applied to denatured chromosomes and hybridisation was carried out under a sealed coverslip overnight in a humidified chamber.

*Probe detection*

After hybridisation slides were washed twice in 50% formamide, 1 × SSC, 42°C, 10 min, followed by two washes in 2 × SSC 42°C, 10 min each. Prior to immunocytochemical detection, slides were incubated with Boehringer Mannheim blocking buffer, (0.5% blocking reagent in 0.1 M maleic acid, 0.15 M NaCl pH 7.5, 0.05% Tween 20), for 30 min at 37°C. The first antibody was anti-digoxigenin raised in sheep (Boehringer Mannheim), 1 $\mu$g ml$^{-1}$, in the above buffer, 37°C, 1 h. Slides were washed for 20 min in 0.1 M maleic acid, 0.15 M NaCl, 0.05% Tween 20, pH 7.5. The second antibody, FITC-conjugated donkey anti-sheep (Jackson ImmunoResearch), diluted in the above blocking buffer at 3 $\mu$g ml$^{-1}$ was applied to slides for 30 min, 37°C. Slides were then washed for 30 min, counterstained with propidium iodide (0.4 $\mu$g ml$^{-1}$) and mounted in anti-fade medium (Vectashield, Vector Labs). Fluorescence was analysed on a Bio-Rad MRC-600 laser scanning confocal microscope equipped with a krypton/argon ion laser using 488/568 nm line excitation and dual channel 522 nm and 585 nm emission filters.

*Immunofluorescence*

Cells were cultured on multi-chamber slides at low cell density, rinsed in PBS and fixed in acetone for 1 min at room temperature. Slides were blocked in 3% BSA in PBS, 0.05% Tween 20 for 10 min prior to the addition of antibodies. Polyclonal antiserum against topoisomerase II alpha was obtained from TopoGen (Columbus Ohio), the monoclonal antibody to topoisomerase II beta was kindly provided by Dr G.C.B. Astaldi Ricotti (Negri *et al.*, 1992). Both antibodies were diluted 1:20 in 3% BSA in PBS, 0.05% Tween. Incubations were for 2 h at room temperature. FITC-conjugated donkey anti-rabbit and FITC-conjugated sheep anti-mouse antibodies (Jackson Immunoresearch), at 30 $\mu$g ml$^{-1}$ in 3% BSA, PBS, 0.05% Tween 20, were applied for 30 min at room temperature to detect the polyclonal alpha antiserum and the monoclonal beta antibody respectively. Slides were washed for 15 min in PBS, 0.05% Tween 20, counterstained, mounted and analysed by confocal microscopy as described above. In order to gain quantitative, comparative data, image acquisition parameters were set and remained unaltered during image collection by confocal microscopy. Pre-incubation of the alpha polyclonal antiserum with its complimentary peptide (TopoGen, Columbus Ohio) abolishes antigen binding and can be used to calibrate the image detection parameters.

*Topoisomerase II assays*

Topoisomerase activity was extracted from cell lines as described by van der Zee *et al.* (1991). Topoisomerase II activity was quantified using a kit obtained from TopoGen (Columbus, Ohio). Decatenation assays were carried out according to the manufacturer's instructions. Inhibition of topoisomerase II activity by etoposide was carried out by addition of the drug to the decatenation assays in 2 $\mu$l of DMSO, (final concentration of DMSO is 2%, also added to controls). Assay products were resolved by agarose gel electrophoresis and stained with 0.5 $\mu$g ml$^{-1}$ ethidium bromide. The gels were then photographed and the negatives contact printed.

**Figure 1** Southern blot analysis of DNA extracted from CALU3 cell line. Lane 1; DNA extracted from SK-MES-1. Lane 2; DNA extracted from L-DAN. Lane 3: DNA extracted from CALU3. All DNA samples were digested with PstI. **a**, Hybridisation to topoisomerase II α, **b**, Hybridisation to RARα, **c**, Hybridisation to PKCα, **d**, Hybridisation to immunoglobulin heavy chain sequences to control for DNA loading.

*Western blot analysis*

Cell extracts containing topoisomerase II activity were resolved on 6% polyacrylamide gels and transferred to Immobilon-P membranes. To detect topoisomerase II alpha by Western blot analysis a polyclonal antibody obtained from Cambridge Research Biochemicals (Cheshire, UK) was used as previously described (Keith *et al.*, 1993). Densitometry was performed using a Molecular Dynamics laser scanning densitometer.

*Southern blot analysis*

Genomic DNA was isolated from cell lines as previously described (Keith *et al.*, 1992), and digested with restriction enzymes according to the manufacturer's directories. Hybridisations were carried out as described previously (Keith *et al.*, 1992). The probe SP1 was used to detect topoisomerase IIα sequences (Chung *et al.*, 1989), pHPKC-alpha 7 to detect PKCα (Parker *et al.*, 1986), p63 to detect RARα and pHJi was used as a control for DNA loading (Keith *et al.*, 1992). The RARα and PKCα probes were obtained from the ATCC.

### Results

*Detection of RARα and topoisomerase II α gene amplification by Southern blot analysis*

Previously, we have shown ERBB2 and topoisomerase IIα to be co-amplified in CALU3 (Keith *et al.*, 1992). We have now expanded this analysis to a number of other chromosome 17q loci.

DNA extracted from CALU3 cell line was analysed for amplifcation of genes on chromosome 17q. Figure 1 shows a Southern blot of DNA extracted from the lung cancer cell lines, SK-MES (lane 1); L-DAN (lane 2); CALU3 (lane 3). The filter was sequentially hybridised to analyse the copy number of (a) topoisomerase IIα; (b) RARα; (c) Protein kinase Cα (PKCα); (d) pHJi loading control. The topoisomerase IIα and RARα loci have previously been localised to chromosome 17q 21-22 (Tsai-Pflugfelder *et al.*, 1988; Mattei *et al.*, 1988). The PKCα locus has been mapped to chromosome 17q 22–24, (Parker *et al.*, 1986). Figure 1 shows that in addition to the topoisomerase IIα locus the RARα locus is amplified in CALU3. Hybridisation of the filter to detect PKCα shows that the increased copy number of RARα and topoisomerase IIα is not due to aneuploidy but to amplification, as CALU3 shows no evidence of increased copy numbers of PKCα sequences. Densitometry of the autoradiographs in Figure 1 shows topoisomerase IIα and RARα sequences to be amplified four fold in CALU3 compared to the other lines.

*Analysis of topoisomerase II alpha amplification by fluorescence* in situ *hybridisation (FISH)*

In order to further characterise the nature of amplified topoisomerase IIα sequences in CALU3 we have used fluorescence *in situ* hybridisation. As shown in Figure 2a, hybridisation of the topoisomerase II alpha cosmid to chromosomes prepared from control lymphocytes detects the two alleles as expected. In the metaphase spread shown all four chromatids are labelled. Figure 2b shows hybridisation of topoisomerase II alpha to a metaphase spread prepared from CALU3. Fluorescent signal is observed on several chromosomes with a cluster of signals localised to one chromosome suggesting that the amplified sequences are intra-chromosomal. There was no evidence for extra-chromosomal sequences. The pattern of hybridisation was consistent between nuclei within the cell line. Each nucleus had five copies of topoisomerase IIα, three of which were clustered on one chromosome. Occas-

**Figure 2** Detection of topoisomerase II alpha gene copies by FISH in **a**, metaphase chromosomes from normal lymphocytes and **b**, metaphase chromosomes from CALU3 adenocarcinoma cell line. Chromsomes counterstained red, alpha gene hybridisation green. Arrows in **b**, show sites of alpha gene copies.

Figure 3 Detection of topoisomerase II alpha expression by Western blot analysis. Lane 1, L-DAN. Lane 2, CALU3, Lane 3, SK-MES. Fifty micrograms of protein were run per lane. Molecular mass standards in kiloDaltons are shown to the right of the autoradiograph.

sionally cells were found with seven regions of hybridisation of which the two additional hybridisations may represent a low level of hybridisation to telomeric repeat sequences. The regions of hybridisation marked by arrows in Figure 2b represent topoisomerase II alpha sequences in CALU3, the remaining two regions of hybridisation are telomeric and due to the complex karyotype of the cell line, cannot be unambiguously assigned as topoisomerase II alpha as the probe used contains repeat sequences which can hybridise to telomeric sequences. However, under the conditions of hybridisation used, the intensity of any telomeric hybridisation in lymphocyte controls is much lower than the topoisomerase IIα signal (data not shown).

*Analysis of topoisomerase II expression by Western blot and immunofluorescence*

The CALU3 cell line is relatively sensitive to topoisomerase II inhibitory agents etoposide and doxorubicin when compared to other lung cancer cell lines (see Materials and methods, Merry *et al.*, 1987; Carmichael *et al.*, 1985; Giaccone *et al.*, 1992). In order to investigate whether expression of the amplified topoisomerase II alpha gene is responsible for the sensitivity to topoisomerase inhibitors, expression of the alpha and beta genes was examined by Western blot analysis and immunofluorescence. For comparison with CALU3, two other non-small cell lung cancer cell lines, L-DAN and SK-MES, which do not have amplified topoisomerase II genes (Keith *et al.*, 1992) were included in the analysis. Figure 3 shows a Western blot of topoisomerase II alpha expression in L-DAN (lane 1), CALU3 (lane 2) and SK-MES (lane 3). From Figure 3 it can be seen that CALU3 expresses topoisomerase II alpha at higher levels than the other cell lines. Densitometric analysis of the autoradiographs shows CALU3 to express 10–15 times more topoisomerase II alpha than the other lines. Therefore amplification of the topoisomerase II alpha gene in CALU3 is accompanied by high levels of expression.

The high levels of topoisomerase II alpha expression in CALU3 was confirmed by immunofluorescence using a second polyclonal antibody as described in the Materials and methods. Figure 4a, c, and e, shows topoisomerase II alpha expression in optical section of L-DAN, CALU3 and SK-MES respectively. The nuclei are counterstained red with propidium iodide and the topoisomerase II alpha expression in CALU3, as visualised by the green fluorescence, is confined to the nucleus as expected. In contrast to Western blot analysis, immunofluorescent detection of the alpha protein in CALU3 highlights the heterogeneity in alpha expression between cells (Figure 4c). Topoisomerase II alpha expression is known to be cell cycle regulated but whether the observed heterogeneity in expression is due to this or sub-population diversity is untested. The lower levels of topoisomerase II alpha expression observed in L-DAN and SK-MES by Western blot analysis were also confirmed by immunofluorescence. However, in order to maintain the optimal conditions for visualisation of the alpha protein in CALU3, alpha expression in the other two lines shown in Figure 4a and e is almost undetectable due to the settings for digital image analysis used for this experiment (see Materials and methods).

The topoisomerase II beta enzyme is also a target for topoisomerase interactive drugs (Drake *et al.*, 1989). We therefore analysed the three cell lines for beta expression to complete their topoisomerase II isoenzyme profiles. We were unable to generate consistent results on beta expression by Western blot analysis due to the instability of the protein during extraction. The instability of the beta protein has been reported on several occasions (Woessner *et al.*, 1990; Negri *et al.*, 1992; Holden *et al.*, 1992). However, there were no major differences in the levels of the 150 kD protein between the three cell lines. There is no information on how the $\beta$ protein degrades and so different cell lines may show varying stability of the $\beta$ protein. In order to circumvent this problem, beta expression was analysed by immunofluorescence. Figure 4b, d and f shows expression of topoisomerase II beta in L-DAN, CALU3 and SK-MES respectively. Beta expression is reported to be largely confined to the nucleolus (Negri *et al.*, 1992) and the data presented in Figure 4 confirms this. All three lines express the beta isozyme and it is expressed in every cell in the population. Immunocytochemistry is only semi-quantitative and due to the intense localised fluorescence pattern obtained when analysing expression of the beta protein, differences in expression levels between the three lines is difficult to assess. Figure 4e shows that using the polyclonal antiserum against the alpha protein, low levels of fluorescence can be detected in the nucleoli. Whether this represents a weak cross-reactivity of this antiserum with the beta protein or co-localisation of the alpha enzyme is not known.

*Biochemical analysis of topoisomerase II activity*

In order to determine whether the high levels of topoisomerase II alpha protein observed in CALU3 has enzyme activity, decatenation assays were carried out. Topoisomerase II activity can be measured due to its ability to monomerise covalently linked kinetoplast DNA circles. Decatenation of kinetoplast DNA requires both the DNA breakage and strand passage activities of topoisomerase II. The decatenation assay does not however distinguish between the alpha and beta isoforms. Figure 5 shows that CALU3 has at least eight-fold more topoisomerase II activity than the other two

**Figure 4** Detection of topoisomerase II alpha and beta expression by immunofluorescence **a** and **b**, L-DAN, **c** and **d** CALU3, **e** and **f** SK-MES. Topoisomerase II alpha expression is shown in **a**, **c** and **e**. Topoisomerase II beta expression **b**, **d** and **f**. Nuclei and nucleoli are counterstained red, topoisomerase II expression is green. Scale bar is 10 microns.

cell lines. L-DAN and SK-MES have almost equivalent topoisomerase II activities. The extracts used in the decatenation assays shown in Figure 5 are the same as those used for the Western analysis of topoisomerase II alpha expression shown in Figure 3. The results of both analyses are in agreement with each other and show CALU3 to express high levels of topoisomerase II. Western blot analysis of the extracts used in the biochemical analysis for beta expression detected degraded beta protein of 150 kD (data not shown). The 150 kD breakdown product of the beta enzyme has been observed previously and shown to be active in the biochemical assays (Negri *et al.*, 1992). Thus, some of the topoisomerase II activity detected may be due to the $\beta$ isoform.

The topoisomerase II activity in protein extracts from CALU3 is largely due to alpha enzyme expression (Figures 3, 4 and 5). The alpha gene is amplified in CALU3 (Figures 1 and 2) and so it is possible that it might contain genetic alterations rendering its protein insensitive to inhibition by topoisomerase II interactive drugs (Takano *et al.*, 1992). We therefore examined the ability of the topoisomerase II interactive drug etoposide to inhibit topoisomerase II activity in decatenation assays. Figure 6 shows that etoposide can inhibit topoisomerase II activity in extracts from all three cell lines, including CALU3. Due to the higher levels of topoisomerase II activity in CALU3, only 1.25 $\mu$g of extract was used per reaction in comparison to 2.5 $\mu$g for L-DAN and SK-MES.

### Discussion

The adenocarcinoma cell line CALU3 has the co-amplification of topoisomerase II alpha and ERBB2 sequences characteristic of a subset of breast adenocarcinomas (Keith *et al.*, 1992, 1993). We therefore examined CALU3 as a suitable model for the study of expression of topoisomerase II alpha from an amplified locus and its consequences for cellular sensitivity to topoisomerase II inhibitory drugs. The

**Figure 5** Decatentation of kinetoplast DNA by cellular extracts from **a**, L-DAN; **b**, CALU3; **c**, SK-MES. Serial dilutions of cellular extracts were assayed for topoisomerase II activity. Lane 2, 2.5 μg; Lane 3, 1.25 μg; Lane 4, 0.625 μg; Lane 5, 0.312 μg; Lane 6, 0.156 μg; Lane 7, 0.078 μg; Lane 8, 0.039 μg; Lane 1, 0 μg. In the absence of cellular extract, catenated kinetoplast DNA, (k), remains in the well of the gel (Lane 1). Topoisomerase II activity decatenates kinetoplast DNA to monomer mini-circles (m).

amplified topoisomerase II alpha gene in CALU3 is expressed at high levels as determined by both Western blot analysis and immunofluorescence, (Figures 3 and 4). Two distinct antibodies were used to examine alpha expression by Western and immunofluorescence therefore confirming by independent methods that the isoform detected is indeed the alpha product. Neither of the other two cell lines tested had such high levels of alpha expression (Figures 3 and 4). CALU3 has five copies of the topoisomerase IIα gene (Figure 2b) yet a 10–15-fold increase in expression over the other two cell lines. This suggests there may be further complexity in the regulation of the topoisomerase IIα gene at the transcriptional or post-transcriptional level.

The human genome has the potential to express a second topoisomerase II isozyme encoded by the topoisomerase II beta gene. There is still a paucity of data on the beta enzyme but since its cloning, recent chromosomal mapping and the generation of monoclonal antibodies against it, progress should now become more rapid (Jenkins *et al.*, 1992; Negri *et al.*, 1992). It is known however that the beta enzyme is expressed, sensitive to inhibition by topoisomerase II interactive drugs and likely to be confined to the nucleolus (Jenkins *et al.*, 1992; Drake *et al.*, 1989; Negri *et al.*, 1992). Any model system used to study topoisomerase II inhibitors would therefore benefit from the analysis of both alpha and beta expression. In contrast to the alpha locus in CALU3, none of the three lung cancer cell lines used in this study have gross chromosomal changes at the beta locus (Keith *et al.*, 1992). By immunofluorescence there is little detectable difference in beta expression between the cell lines, with all lines expressing the beta isozyme in every cell (Figure 4). The localisation of the beta product to the nucleoli confirms the data of Negri *et al.*, 1992 and suggests a role for the beta product in ribosomal gene expression or organisation (Kim & Wang, 1989). The data presented in Figure 4 on beta expression suggest that the beta product represents a target for inhibition and may be a target for topoisomerase II poisons in the absence of alpha isozyme expression.

In addition to the correlation between alpha gene amplification in CALU3 and high levels of alpha expression, biochemical assays for topoisomerase II activity confirmed a concomitant elevated level of activity which could be inhibited by etoposide (Figures 4 and 5). Taken together, the Western blot analysis, immunofluorescence studies and the enzyme activity assays suggest that the sensitivity of CALU3 to topoisomerase inhibitors (Merry *et al.*, 1987) is due to an amplified and over-expressed topoisomerase II alpha gene. The finding that in CALU3, expression of an amplified topoisomerase II alpha gene can confer relative sensitivity to the cytotoxic effects of topoisomerase II inhibitors is of

**Figure 6** Inhibition of topoisomerase II activity by etoposide. Cell extracts from **a**, L-DAN; **b**, CALU3; **c**, SK-MES were assayed for decatenation activity in the presence of increasing concentrations of etoposide, Lane 1, no drug, no protein extract; Lane 2, DMSO dilutant control; Lane 3, 10 μM; Lane 4, 100 μM; Lane 5, 200 μM. Lanes 2 to 5 in **a** and **c** were assays using 2.5 μg protein, Lanes 2 to 5 in **b** was an assay using 1.25 μg of CALU3 extract. To the right of the figure the catenated kinetoplast DNA (k) and the decatenated monomer mini-circles (m) are marked.

importance to tumours where topoisomerase II alpha gene amplification has occurred, as these cases may benefit preferentially from treatment with topoisomerase II inhibitors.

We have previously shown that the region of amplification found in primary breast tumours encompasses at least three loci including ERBB2, RARα and topoisomerase IIα. The PKCα locus was found to be outside the region of amplification showing that the increased copy number of the three genes is due to amplification rather than aneuploidy (Keith *et al.*, 1993). We have also previously shown that the lung adenocarcinoma cell line, CALU3, has co-amplification ERBB2 and topoisomerase IIα (Keith *et al.*, 1992). As part of our efforts to characterise similarities between the genetic events found around the topoisomerase IIα locus in breast cancer biopsies and the CALU3 cell line, we have shown that the RARα locus is also amplified in CALU3 (Figure 1). By Southern analysis, topoisomerase IIα and RARα are amplified around 4-fold. The finding that the locus for PKCα is not within the amplicon, is again consistent with the observations in breast cancer (Keith *et al.*, 1992) and demonstrates that the increased copy number of ERBB2, RARα and topoisomerase IIα in CALU3 is not due to aneuploidy. The RARα receptor regulates normal cellular proliferation and differentiation through activation by retinoids, (Bollag & Holdener, 1992) and can, when expressed as a fusion protein in acute promyelocytic leukaemia act as an oncogene. (Clarkson, 1991). Gebert *et al.* (1991) have shown the RARα gene to be expressed at high levels in CALU3 and so CALU3 may be a suitable model to study retinoids in cancer therapy, (Bollag & Holdener, 1992).

The finding that topoisomerase II alpha can be amplified and expressed in adenocarcinomas opens the question as to whether there is intra-tumour heterogeneity in topoisomerase IIα gene amplification and its consequences for the sensitivity of subpopulations within the tumour to cytotoxic agents. Fluorescence *in situ* hybridisation is an ideal approach to study this heterogeneity. Indeed, it has recently been shown by FISH that in breast cancer biopsies there is considerable intra-tumour variation in ERBB2 copy number (Kallioniemi *et al.*, 1992). Our demonstration that amplification of topoisomerase II alpha can be studied by FISH (Figure 2), opens up the possibility of examining the importance of topoisomerase gene amplification and allele imbalance at the single cell level.

The authors would like to thank Professor P. Workman for stimulating discussions, Dr P. Rabbitts and Dr A. Heppell-Parton for the introduction to FISH and to Dr G. Astaldi Ricotti for the topoisomerase IIβ antibodies. We would also like to thank Dr I. Hickson for his generous gift of the topoisomerase IIα cosmid. This work was supported by the Cancer Research Campaign.

### References

ANDERSON, H. & ROBERGE, M. (1992). DNA topoisomerase II: A review of its involvement in chromosome structure, DNA replication, transcription and mitosis. *Cell Biol. Internat. Rep.*, **16**, 717-724.

BOLLAG, W. & HOLDENER, E.E. (1992). Retinoids in cancer prevention and therapy. *Ann. Oncol.*, **3**, 513-526.

CARMICHAEL, J., MITCHELL, J.B., DEGRAFF, W.G., GAMSON, J., GAZDAR, A.F., JOHNSTON, B.E., GLATSTEIN, E. & MINNA, J.D. (1985). Chemosensitivity testing of human lung cancer cell lines using the MTT assay. *Br. J. Cancer*, **57**, 540-547.

CHUNG, T.D.Y., DRAKE, F.H., TAN, K.B., PER, S.T., CROOKE, S.T. & MIRABELLI, C.K. (1989). Characterisation and immunological identification of cDNA clones encoding two human DNA topoisomerase II isoenzymes. *Proc. Natl Acad. Sci. USA*, **86**, 9431-9435.

CLARKSON, B. (1991). Retinoic acid in acute promyelocytic leukaemia: the promise and the paradox. *Cancer Cells*, **3**, 211-220.

DAVIES, S.M., ROBSON, C., DAVIES, S.L. & HICKSON, I.D. (1988). Nuclear topoisomerase II levels correlate with the sensitivity of mammalian cells to intercalculating agents and epipodophyllotoxins. *J. Biol. Chem.*, **263**, 17724-17729.

DRAKE, F.H., HOFMANN, G.A., BARTUS, H.F., MATTERN, M.R., CROOKE, S.T. & MIRABELLI, C.K. (1989). Biochemical and pharmacological properties of p170 and p180 forms of topoisomerase II. *Biochemistry*, **28**, 8154-8160.

GEBERT, J.F., MOGHAL, N., FRANGIONI, J.V., SUGARBAKER, D.J. & NEEL, B.G. (1991). High frequency of retinoic acid receptor β abnormalities in human lung cancer. *Oncogene*, **6**, 1859-1868.

GIACCONE, G., GAZDAR, A.F., BECK, H., ZUNINO, F. & CAPRANICO, G. (1992). Multidrug sensitivity phenotype of human lung cancer cells associated with topoisomerase II expression. *Cancer Res.*, **52**, 1666-1674.

HOCHHAUSER, D., STANWAY, C.A., HARRIS, A.L. & HICKSON, I.D. (1992). Cloning and characterisation of the 5'-flanking region of the human topoisomerase II α gene. *J. Biol. Chem.*, **267**, 18961-18965.

HOLDEN, J.A., ROLFSON, D.H. & WITTWER, C.T. (1992). The distribution of immunoreactive topoisomerase II protein in human tissues and neoplasms. *Oncol. Res.*, **4**, 157-166.

JENKINS, J.R., AYTON, P., JONES, T., DAVIES, S.L., SIMMONS, D.L., HARRIS, A.L., SHEER, D. & HICKSON, I.D. (1992). Isolation of cDNA clones encoding the $\beta$ isozyme of human DNA topoisomerase II and localisation of the gene to chromsome 3p24. *Nucleic Acids Res.*, **20**, 5587–5592.

KALLIONIEMI, O.P., KALLIONIEMI, A., KURISA, W., THOR, A., CHEN, L.-C., SMITH, H.S., WALDMAN, F.M., PINKEL, D. & GRAY, J.W. (1992). ERBB2 amplification in breast cancer analysed by fluorescence *in situ* hybridisation. *Proc. Natl Acad. Sci. USA*, **89**, 5321–5325.

KEITH, W.N., DOUGLAS, F., WISHART, G.C., MCCALLUM, H.M., GEORGE, W.D., KAYE, S.B. & BROWN, R. (1993). Co-amplification of ERBB2, topoisomerase II $\alpha$ and retinoic acid receptor $\alpha$ genes in breast cancer and allelic loss at topoisomerase I on chromosome 20. *Eur. J. Cancer*, (in press).

KEITH, W.N., TAN, K.B. & BROWN, R. (1992). Amplification of the topoisomerase II $\alpha$ gene in a non-small cell lung cancer cell line and characterisation of polymorphisms at the human topoisomerase II $\alpha$ and $\beta$ loci in normal tissue. *Genes, Chromosome & Cancer*, **4**, 169–175.

KIM, R. & WANG, J.C. (1989). A subthreshold level of DNA topoisomerases leads to the excision of yeast rDNA as extrachromosomal rings. *Cell*, **57**, 975–985.

LIU, L.F. (1989). DNA topoisomerase poisons as anticancer drugs. *Ann. Rev. Biochem.*, **58**, 351–375.

MATTEI, M.G., PETKOVICH, M., MATTEI, J.F., BRAND, N. & CHAMBON, P. (1988). Mapping of the human retinoic acid receptor to the q21 band of chromosome 17. *Hum. Genet.*, **80**, 186–188.

MERRY, S., COURTNEY, E.R., FETHERSTON, C.A., KAYE, S.B. & FRESHNEY, R.I. (1987). Circumvention of drug resistance in human non-small cell lung cancer *in vitro* by verapamil. *Br. J. Cancer*, **56**, 401–405.

MUGGIA, F.M. & GILL, P.S. (1991). In *DNA Topoisomerases in Cancer*, Potmesil, M. & Kohn, K.W. (eds), 312–318. Oxford University Press.

NEDERLOF, P.M., VAN DER FLIER, S., RAAP, A.K. & TANKE, H.J. (1992). Quantification of inter and intra-nuclear variation of fluorescence *in situ* hybridisation signals. *Cytometry*, **13**, 831–838.

NEGRI, C., CHIESA, R., CERINO, A., BESTAGNO, M., SALA, C., ZINA, MARALDI, N.M. & ASTALDI RICOTTI, G.C.B. (1992). Monoclonal antibodies to human DNA topoisomerase I and the two isoforms of DNA topoisomerase II; 170 and 180 kDa isozymes. *Exp. Cell. Res.*, **200**, 452–459.

NITISS, J.L., LIU, Y.-X., HARBURG, P., JANNATIPOUR, M., WASSERMAN, R. & WANG, J. (1992). Amsacrine and etoposide hypersensitivity of yeast cells overexpressing DNA topoisomerase II. *Cancer Res.*, **52**, 4467–4472.

PARKER, P.J., COUSSENS, L., TOTTY, N., RHEE, L., YOUNG, S., CHEN, E., STABLE, S., WATERFIELD, M.D. & UURICH, A. (1986). The complete primary structure of protein kinase C – the major phorbolester receptor. *Science*, **233**, 853–859.

PINKEL, D., STRAUME, T. & GRAY, J.W. (1986). Cytogenetic analysis using quantitative, high sensitivity, fluorescence hybridisation. *Proc. Natl Acad. Sci. USA*, **83**, 2934–2938.

PLUMB, J.A., MILROY, R. & KAYE, S.B. (1989). Effects of pH dependence of 3-(4,5-dimethylthiazol-2-yl)-2, 5-diphenyl-tetrazolium bromide – formazan absorption on chemosensitivity determined by a novel tetrazolium based assay. *Cancer Res.*, **49**, 4435–4440.

TAKANO, H., KOHNO, K., MATSUO, K., MATSUDA, T. & KUWANO, M. (1992). DNA topoisomerase targeting antitumour agents and drug resistance. *Anti-Cancer Drugs*, **3**, 323–330.

TAN, K.B., DORMAN, T.E., FALLS, K.M., CHUNG, T.D.Y., MIRABELLI, C.K., CROOKE, S.T. & MAO, J.-I. (1992). Topoisomerase II $\alpha$ and topoisomerase II $\beta$: Characterisation and mapping to human chromosomes 17 and 3 respectively. *Cancer Res.*, **52**, 231–234.

TSAI-PFLUGFELDER, M., LIU, L.F., LIU, A.A., TEWEY, K.M., WHANG-PENG, J., KNUTSEN, T., HUEBNER, K., CROCE, C.M. & WANG, J.C. (1988). Cloning and sequencing of cDNA encoding human DNA topoisomerase II and localisation of the gene to chromosome regions 17q 21–22. *Proc. Natl Acad. Sci. USA*, **85**, 7177–7181.

VAN DER ZEE, A.G.J., HOLLEMA, H., DE JONGS, L.S., BOONSTRA, H., GOUW, A., WILLEMSE, P.H.B., ZILJLSTRA, J.G., DE VRIES, E.G.E. (1991). P-glycoprotein expression and DNA topoisomerase I and II activity in benign tumours of the ovary and in malignant tumours of the ovary, before and after platinum/cyclophosphamide chemotherapy. *Cancer Res.*, **51**, 5915–5920.

WOESSNER, R.D., CHUNG, T.D.Y., HOFMANN, G.A., MATTERN, M.R., MIRABELLI, C.K., DRAKE, F.H. & JOHNSON, R.K. (1990). Differences between normal and *ras* transformed NIH 3T3 cells in expression of the 170 kD forms of topoisomerase II. *Cancer Res.*, **50**, 2901–2908.

WOESSNER, R.D., MATTERN, M.R., MIRABELLI, C.K., JOHNSON, R.K. & DRAKE, F.H. (1991). Proliferation and cell cycle dependent differences in expression of the 170 kilodalton and 180 kilodalton forms of topoisomerase II in NIH 3T3 cells. *Cell Growth & Differentiation*, **2**, 209–214.